# Supplementary figures and images for: Pak1 and PP2A antagonize aPKC function to support cortical tension induced by the Crumbs-Yurt complex
Source: eLife. 2021 Jul 2;10:e67999. doi: 10.7554/eLife.67999 (PMC8282337; doi:10.7554/eLife.67999)

Y1k →  
Sp

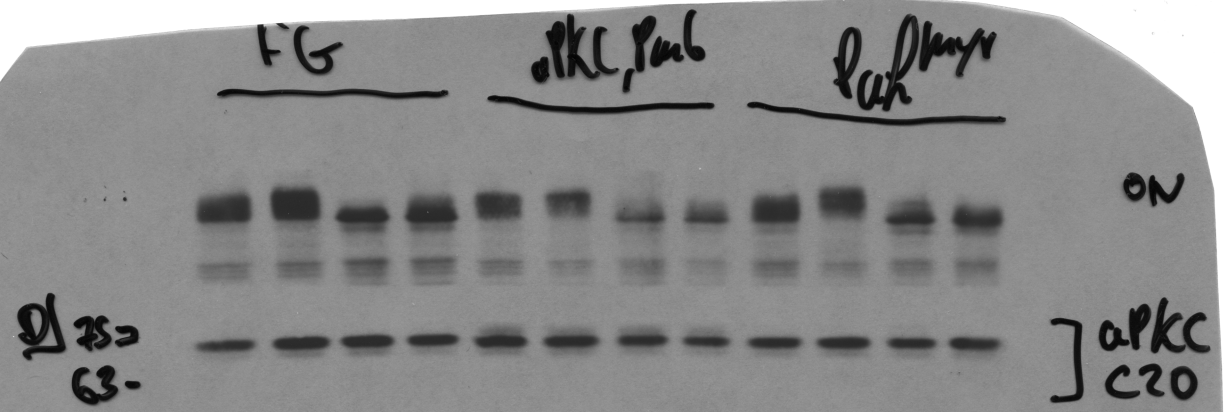

Y1k →  
Sp

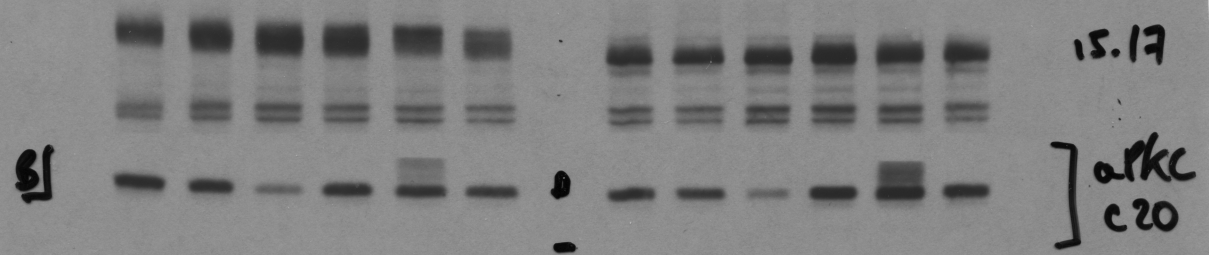

Y1k Sp →

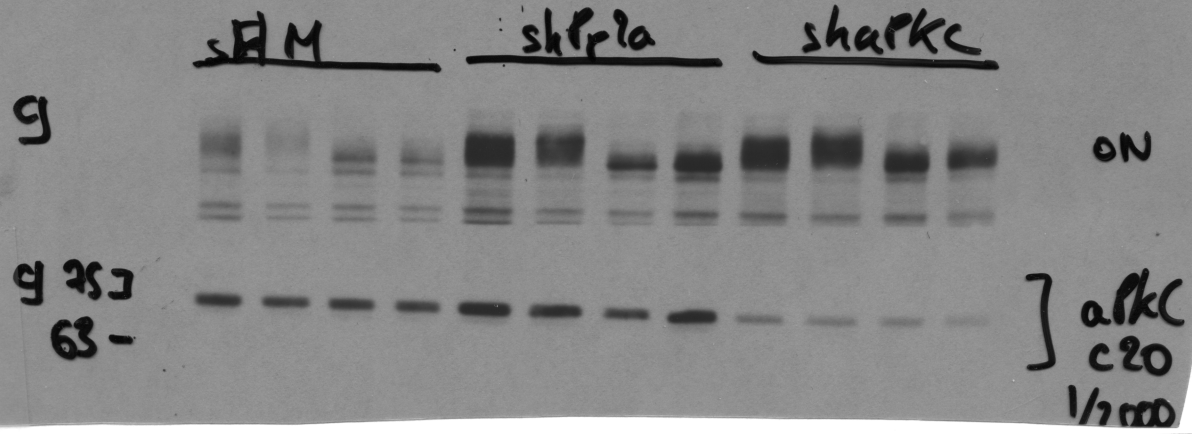

~~exp~~

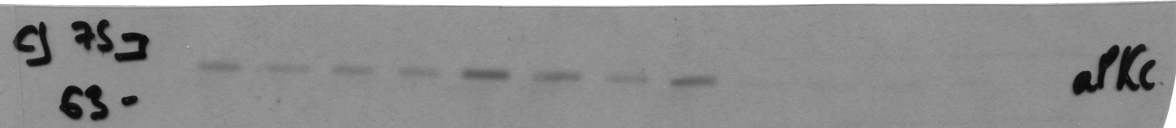

Supplement: Figure 7—source data 2. — Original scan for Figure 7C. [file elife-67999-fig7-data2.pdf]

$\beta$  tub E7 1/2000

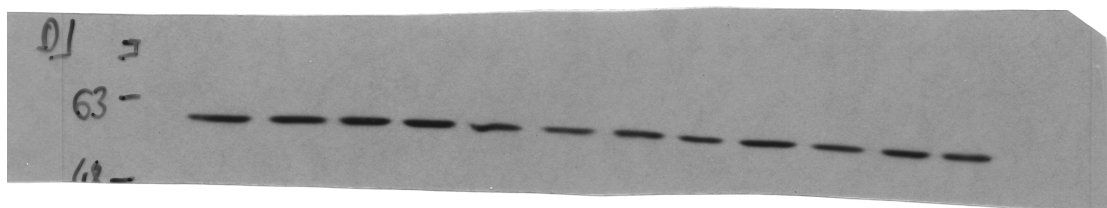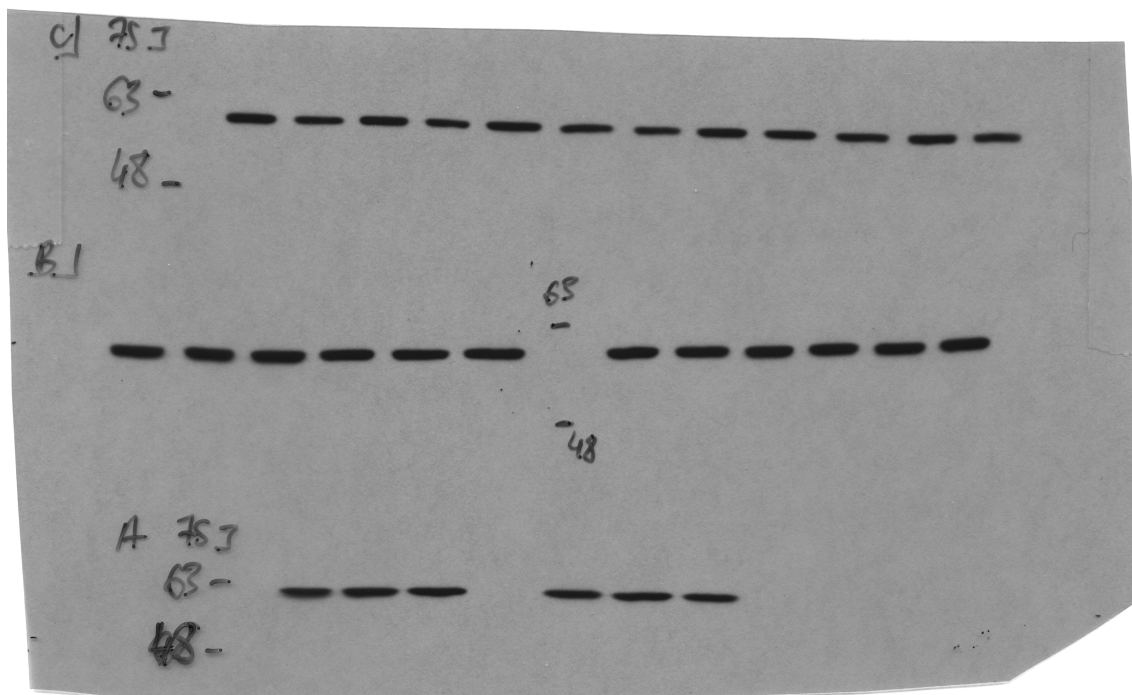

Supplement: Figure 7—source data 5. — Original scan of loading controls. [file elife-67999-fig7-data5.pdf]

Figure 7-C

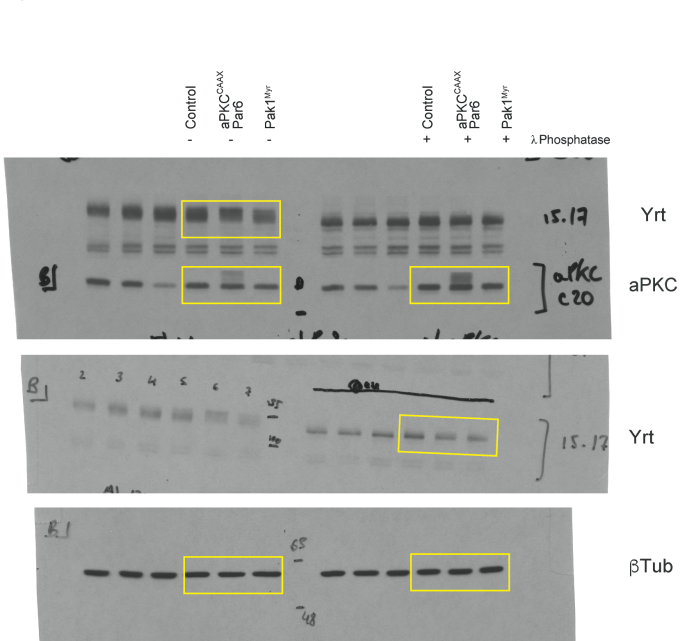

Figure 7-D

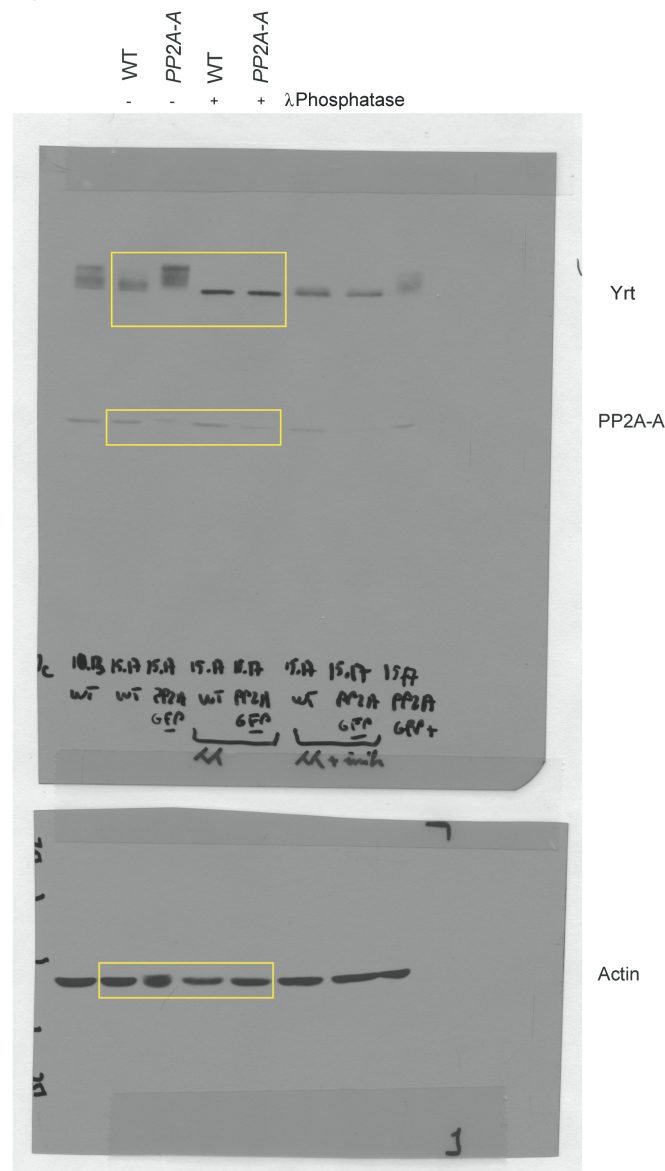

Figure 7-E, F

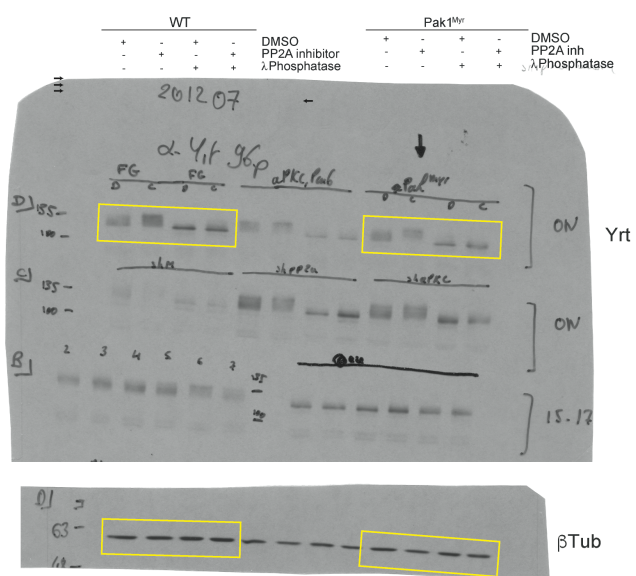

Supplement: Figure 7—source data 6. — Region of each blot shown in Figure 7C–F are highlighted with a yellow box. [file elife-67999-fig7-data6.pdf]
