# Supplementary material for: Pak1 and PP2A antagonize aPKC function to support cortical tension induced by the Crumbs-Yurt complex
Source: eLife. 2021 Jul 2;10:e67999. doi: 10.7554/eLife.67999 (PMC8282337; doi:10.7554/eLife.67999)
Supplement: Figure 7—source data 3. — Original scan for Figure 7D. [file elife-67999-fig7-data3.pdf]

18 Fév 201

Expression de Yurt dans mutant PP2A-A

Triage des ♀ stage 15-17 PP2A-A ponte 4h40 → 16h
Lyse Triton 1% + PMSF + POLA + NEM NaB Nico + TSA
50µg en chaq échantillon
Trait^t λ Phosphatase 1h à 30°C

Dépôt

WT	WT	PP2A	λ Phosph WT	λ Phosph PP2A	λ Pho + [illegible] WT	λ Pho + [illegible] PP2A	PP2A GFP+
10-13	15-17	15-17	15-17	15-17	15-17	15-17	15-17

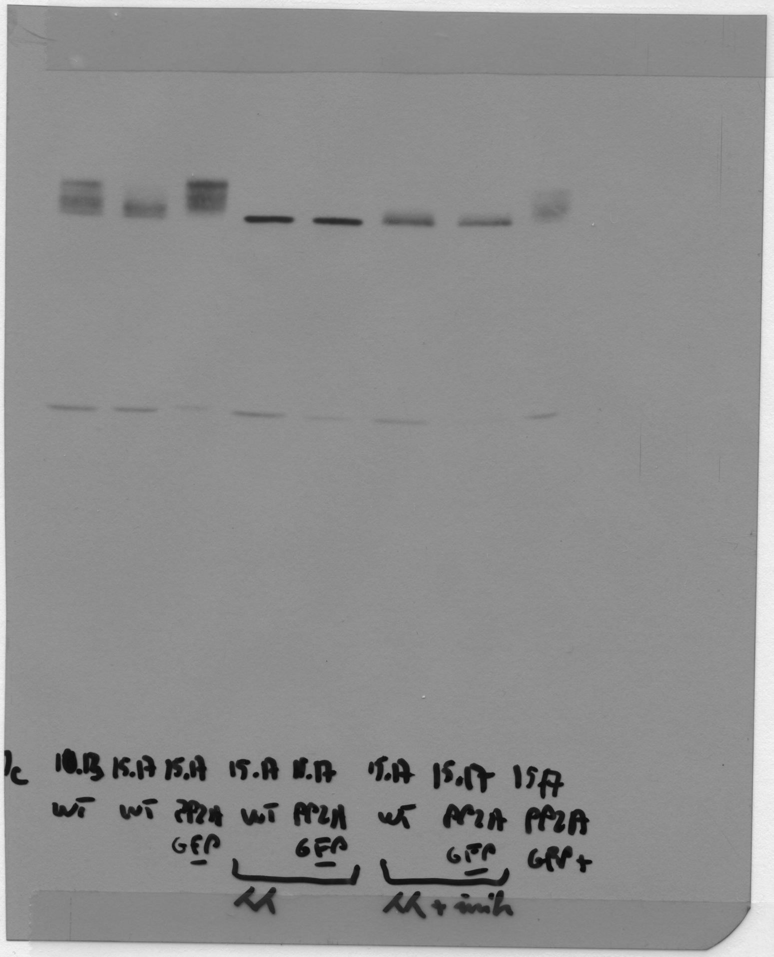

WB Yurt 96 1/10000

WB PP2A 1/5000

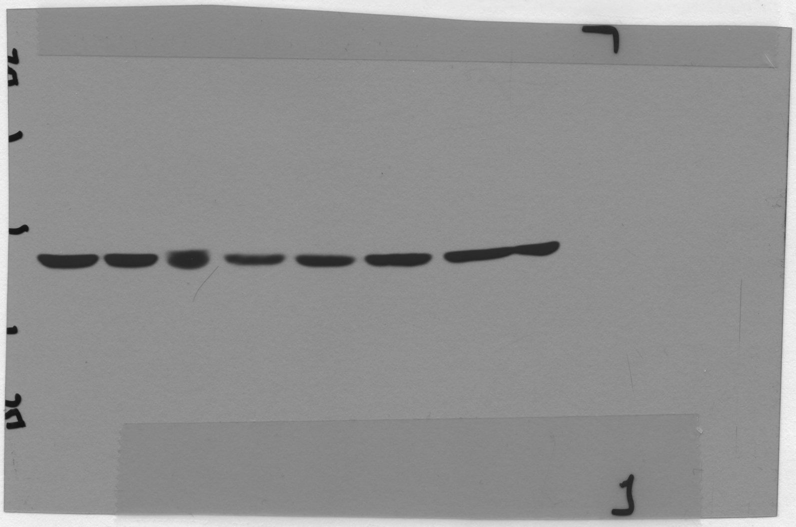

WB Actin 1/2500
