# Supplementary material for: Pak1 and PP2A antagonize aPKC function to support cortical tension induced by the Crumbs-Yurt complex
Source: eLife. 2021 Jul 2;10:e67999. doi: 10.7554/eLife.67999 (PMC8282337; doi:10.7554/eLife.67999)

(P)° Yit

15-17 FG, aPKC, Par6, Pak myr OK

st 11-13 ∅ à conclure

Pak myr +/- Canth bean

FG aussi

shPp2a aussi (∅ de [illegible] (P)°

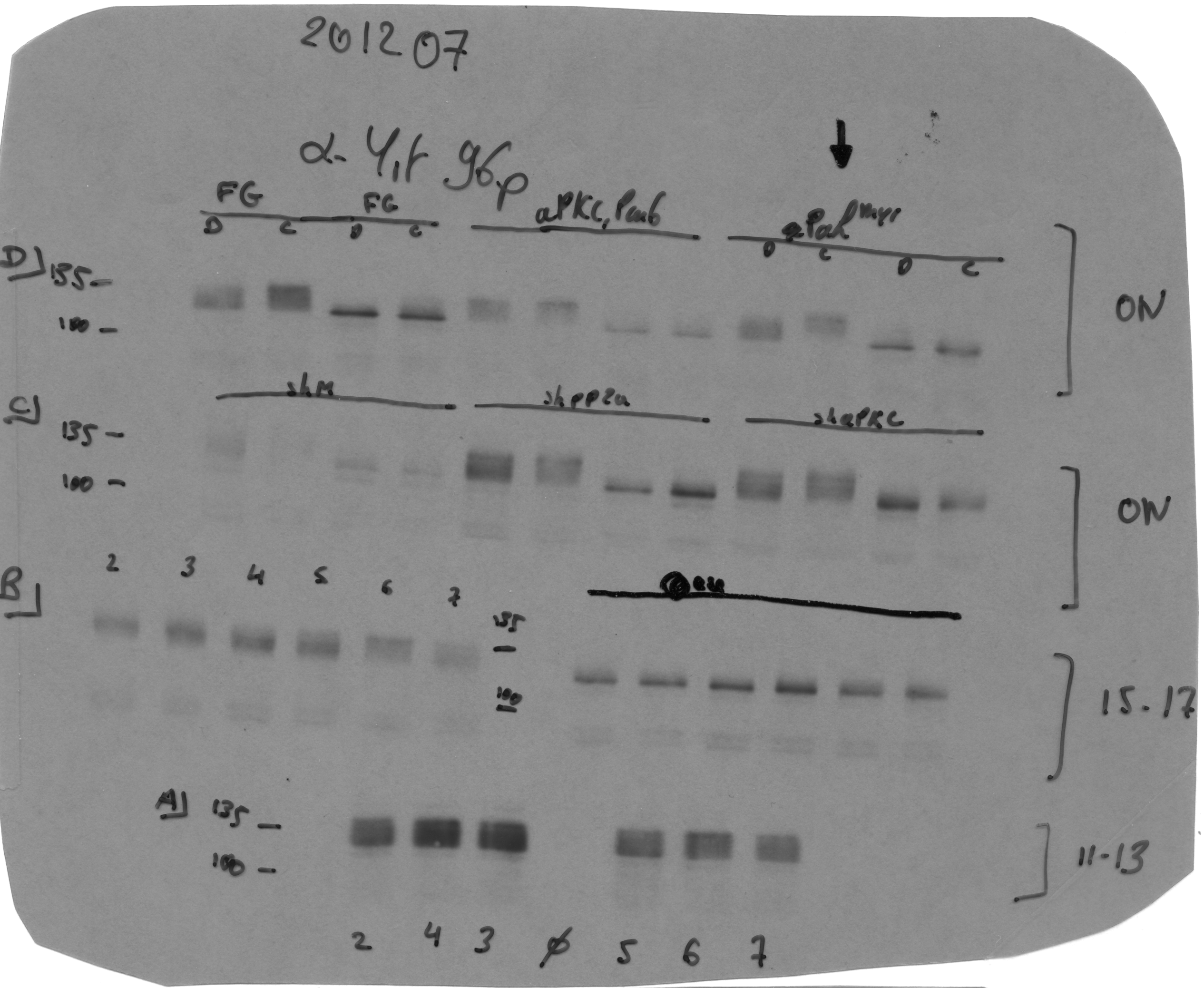

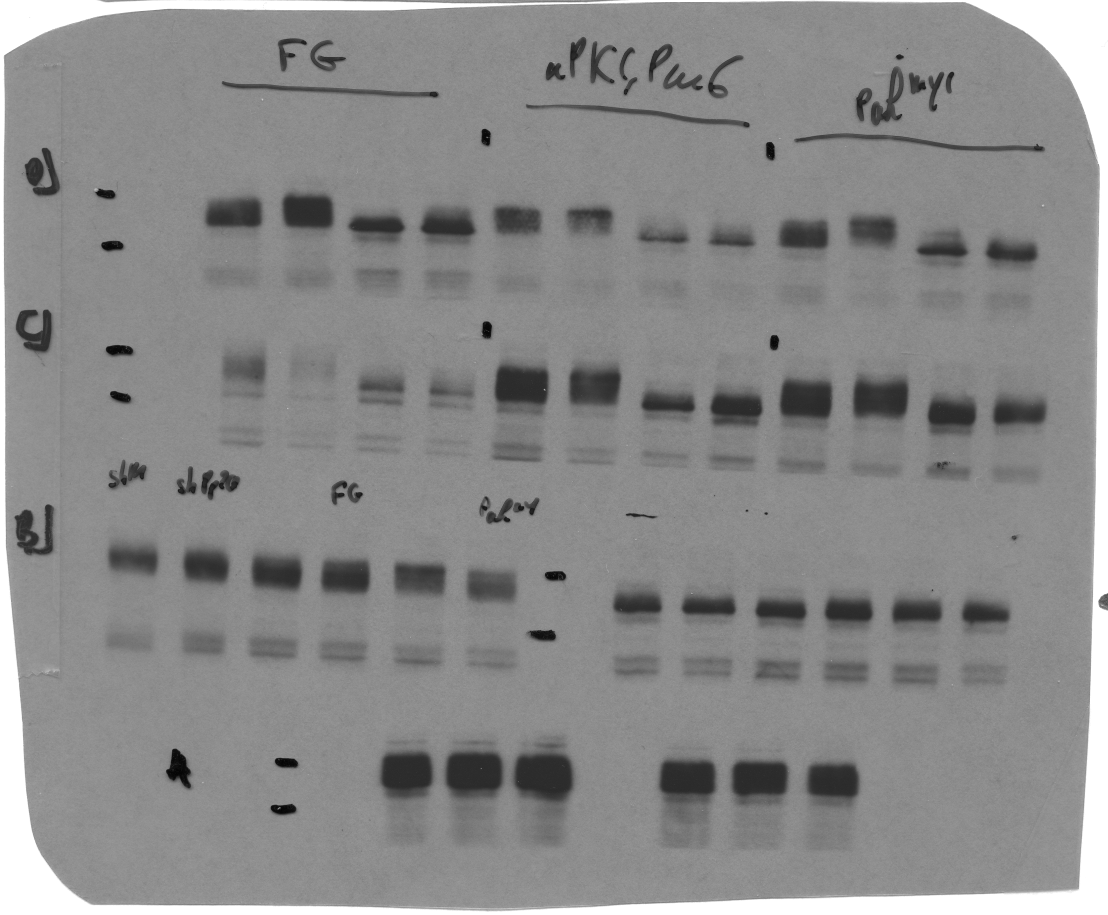

Supplement: Figure 7—source data 4. — Original scan for Figure 7E,F. [file elife-67999-fig7-data4.pdf]
